# Clinical and radiographic characteristics of osteosarcomas of the jaws: A retrospective study

**DOI:** 10.4317/jced.61827

**Published:** 2024-08-01

**Authors:** Mônica-Rezende Veloso, Mariana-Quirino Soares, Andresa-Borges Soares, Francine Panzarella, Monikelly Nascimento, José-Luiz Junqueira

**Affiliations:** 1 DDS, Postgraduate Program in Oral Radiology, São Leopoldo Mandic Research Institute, Campinas, SP, Brazil; 2 Professor, Oral Radiology Division, São Leopoldo Mandic Research Institute, Campinas, SP, Brazil; 3 Oral Pathology Division, São Leopoldo Mandic Research Institute, Campinas, SP, Brazil

## Abstract

**Background:**

Osteosarcomas in the maxillofacial region are rare and present different clinical and epidemiological aspects than those diagnosed in the long bones. This retrospective cross-sectional observational study aimed to report the characteristics of osteosarcomas of the jaws diagnosed in an oral pathology reference laboratory.

**Material and Methods:**

Information (gender, location of origin, age, symptoms, lesion evolution time, and clinical appearance) regarding the cases diagnosed as osteosarcomas between 2001 and 2023 were obtained from histopathology reports, exam request forms, and clinical photographs. All radiographs were evaluated on a high-resolution screen by a previously trained radiologist. A 20-year experienced pathologist reviewed the histopathological slides and determined the predominant matrix of the lesions: osteoblastic, chondroblastic, or fibroblastic.

**Results:**

Seventeen out of 33,692 cases diagnosed by the oral pathology laboratory over 22 years were osteosarcomas and 10 were included for analysis. The majority were diagnosed in males (60%) and the overall mean age was 37.8±21.6 years. A swollen, reddish, and ulcerated area was the most common clinical appearance. The mean evolution time of the lesions was 5.2±6.6 months. The majority of osteosarcomas were histologically classified as osteoblastic (80%). The radiographic appearance of the lesions was predominantly mixed (60%), presenting tooth resorption (44.4%) or displacement (33.3%), pericementum thickening (55.5%), mandibular canal erosion (71.4%) and sunray periosteal reaction (80%).

**Conclusions:**

The osteosarcomas of the jaws are predominantly osteoblastic with a swollen, reddish, and ulcerated clinical appearance. Imaging exams reveal mixed lesions with sunray periosteal reaction.

** Key words:**Oral Pathology, Radiology, Osteosarcoma, Imaging diagnosis.

## Introduction

Although osteosarcoma is a highly prevalent malignant bone tumor, lesions in the jaws represent only 4.4% (1-3). While the lesions in long bones are diagnosed during adolescence in areas of marked bone growth, osteosarcomas of the jaws are usually diagnosed in adults (31 to 39 mean age) after bone maturation and present low metastasis and longer survival rates (3-6).

Although the main cause of osteosarcomas remains uncertain, previous radiotherapy of malignant lesions, Paget’s disease, fibro-osseous lesions, and Li Fraumeni syndrome are well-known risk factors (7-10). The prognosis of these lesions in the jaws depends on tumor grading, metastasis level, recurrence, and possibility of resection (5); however, their complete removal can become difficult due to proximity to important craniofacial structures as well as esthetic and functional demands in the face (6).

Despite the low prevalence of osteosarcomas in the maxillofacial region, clinical, radiographic, and histopathological evidence must be continuously gathered to support further therapeutic approaches (6). Therefore, this study aimed to report the characteristics of osteosarcomas of the jaws diagnosed in an oral pathology reference laboratory.

## Material and Methods

This retrospective cross-sectional observational study, which was approved by the Research Ethics Committee of the São Leopoldo Mandic Faculty (file #61379222.0.0000.5374), selected the cases histopathologically diagnosed as osteosarcomas between 2001 and 2023 in the oral pathology laboratory. The study was reported in accordance with the Enhancing the Quality and Transparency of Health Research” (EQUATOR Network) and the STROBE (The Strengthening the Reporting of Observational Studies in Epidemiology) checklist (11).

The cases with low-quality radiographs and/or without histopathological slides were excluded. Only radiographic and clinical images obtained at the diagnosis were considered. Information regarding gender, location of origin, age, symptoms, lesion evolution time, clinical appearance, and diagnosis was obtained from histopathology reports, exam request forms, and clinical photographs. All radiographs were evaluated on a high-resolution screen by a previously trained radiologist. A 10-year experienced radiologist was consulted in case of doubt. The following aspects of the osteosarcomas were observed:

• radiographic appearance: radiolucency, radiopacity, or mixed;

• margins: marked or ill-defined;

• location: maxilla or mandible, anterior or posterior;

• adjacent teeth: normal, resorption, displacement, floating;

• pericementum: normal or thickening;

• mandibular canal: normal, displacement, or erosion;

• cortical bone: normal, erosion, expansion, or thinning;

• periosteal reaction: absent, lamellar, or sunray.

A 20-year experienced pathologist reviewed the histopathological slides and determined the predominant matrix of the lesions: osteoblastic, chondroblastic, or fibroblastic. For the radiographic analysis, cases were reevaluated after 30 days and a 0.93 (95% CI: 0.75-1.0) intraobserver Kappa agreement was observed. The absolute and relative frequencies were computed for descriptive data analysis (SPPS 26, IBM, Chicago, IL, USA).

## Results

Seventeen out of 33,692 cases diagnosed by the oral pathology laboratory over 22 years were osteosarcomas (0.05% prevalence). Only 10 cases presented images with satisfactory quality and histopathological slides for adequate evaluation.

**Figure 1:**
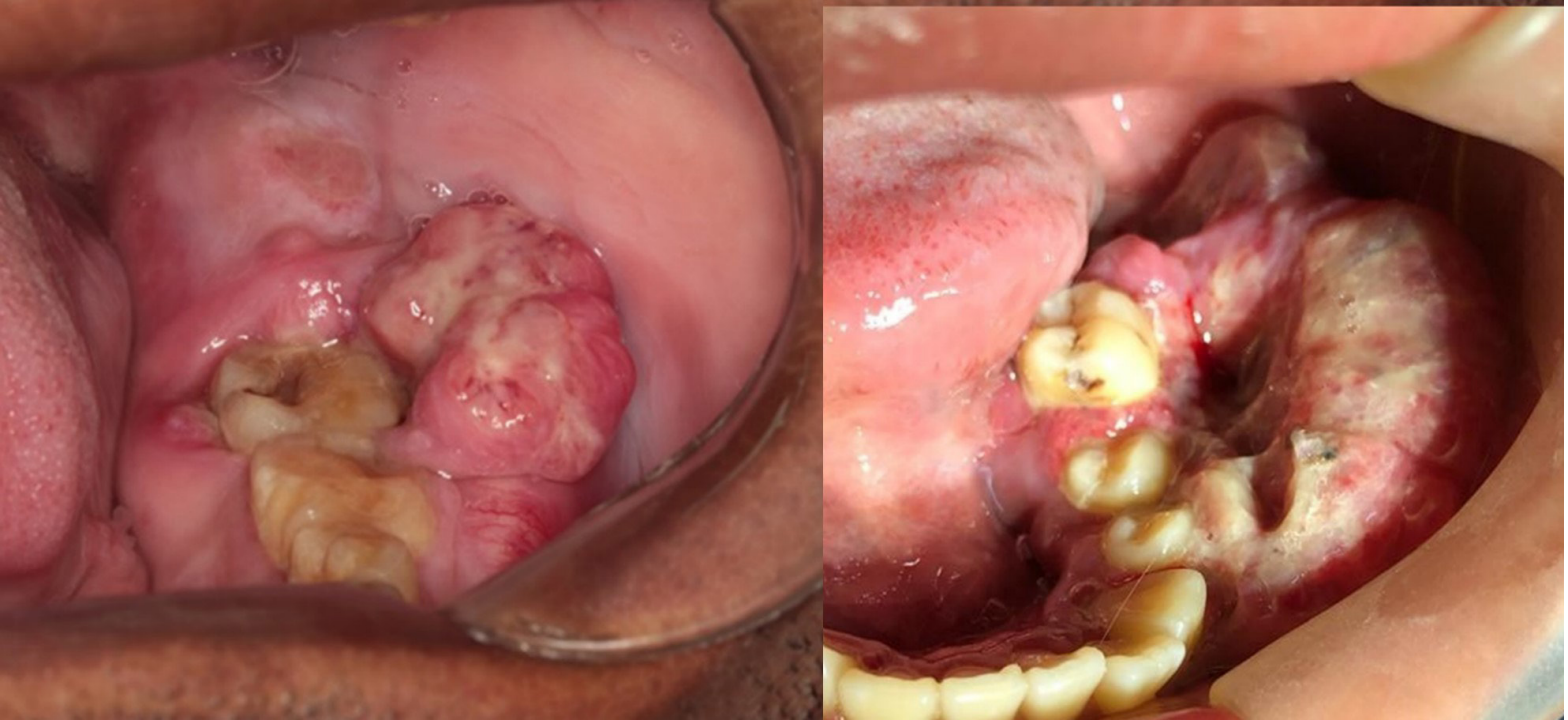
Swollen, reddish, and ulcerated areas in the posterior mandible of two patients.

**Figure 2:**
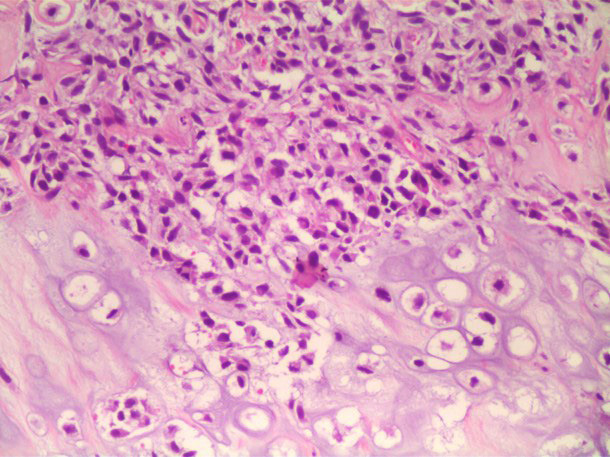
Hematoxylin and eosin-stained histopathological slide of osteosarcoma with cartilage areas and atypical cells (arrow), osteoid material (asterisk), pleomorphic cells, and nuclear hyperchromatism (200x magnification).

Sixty and 40% of osteosarcomas were respectively diagnosed in males and females, aged from 13 to 81 years (mean 37.8±21.6). Nine patients were Brazilians while one patient originated from Angola. Except for one case that was not reported, the other osteosarcomas were submitted to incisional biopsy. Four patients reported pain and 70% of the osteosarcomas affected the mandible. A swollen, reddish, and ulcerated area was the most common clinical appearance (70%) (Fig. 1). The evolution time of the lesions varied from 1 to 21 months (mean 5.2±6.6). The majority of osteosarcomas were histologically classified as osteoblastic ( Table 1, Fig. 2).

**Figure 3:**
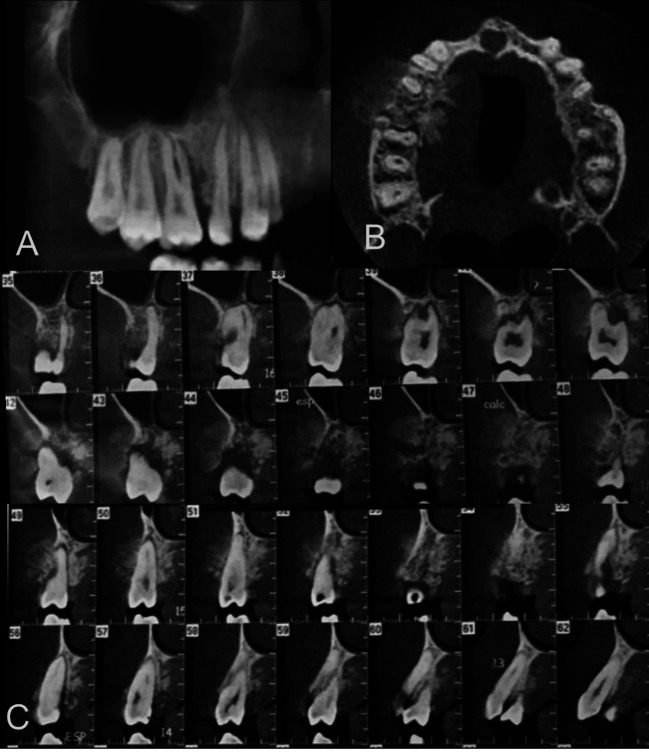
Tomographic image of an osteosarcoma. (A) Panoramic, (B) axial, and (C) parasagittal reconstructions. Mixed lesion with ill-defined margins in the region of right maxillary molars and premolars, irregular pericementum thickening, erosion of the cortical bones, and sunray periosteal reaction.

The radiographic appearance of the lesions was predominantly mixed (60%), followed by radiopacity (30%). All osteosarcomas presented ill-defined margins. Except for one case without adjacent teeth, the majority of osteosarcomas caused tooth resorption (44.4%) or displacement (33.3%). The adjacent teeth were not affected in two cases. The pericementum was thickened in six cases (55.5%). The mandibular canal erosion (resorption of the cortical) was the predominant aspect among the lesions in the posterior mandible (71.4%). The mandibular canal was found normal in one case and displaced by other osteosarcoma. The cortical bone of the maxilla or mandible was eroded in the majority of cases (70%). The sunray effect was the predominant periosteal reaction (80%) ( Table 2,Fig. 3).

## Discussion

The malignant bone neoplasm known as osteosarcoma rarely occurs in the jaws and the possibility of complete removal plays a key role in its prognosis (6). The early diagnosis is essential to allow the resection of osteosarcomas still restricted to the bones without affecting noble adjacent structures (12). Retrospective observational studies represent the main resource for analyzing and comparing the characteristics of these rare lesions. Despite 17 cases were diagnosed as osteosarcomas by an oral pathology laboratory, only 10 cases were included in this study. The wide age range of the patients (between 13 and 81 years) corroborates previous studies that reported osteosarcomas in patients aged from 10 to 87 years (13,14); in addition, osteosarcomas of the jaws were reported in older patients than those diagnosed with osteosarcomas in the long bones (5).

The majority of males diagnosed with osteosarcomas is also in line with the 1.3:1 male/female ratio reported in the literature for osteosarcomas regardless of location (3). However, the diverse ratios reported for osteosarcomas in the craniofacial region may be related to the limited number of patients in each study (12-14).

The painful, swollen, reddish, and ulcerated lesions observed in this study are similar to those reported by Bennet *et al*. (2000) (14). The same authors also reported that the time between the lesion onset and diagnosis mostly varied between 2 to 6 months (mean 4.6) (14), while the lesion evolution time was 2.8 months in this study.

Most osteosarcomas presented a mixed radiographic appearance with ill-defined margins, tooth resorption or displacement, resorption of the mandibular canal lamina dura and cortical bones, and sunray periosteal reaction. Pericementum thickening of adjacent teeth was surprisingly observed in 50% of the cases. Garrington *et al*. (1967) emphasized that osteosarcomas can infiltrate and induce symmetrical thickening of the periodontal ligament before radiographic signs can be visualized (15). It is important to highlight that osteosarcomas do not present homogeneous radiographic characteristics (14,16); however, the findings of this study corroborate the literature regarding predominantly mixed appearance and sunray effect (17,18). Although sunray is considered a pathognomonic aspect of osteosarcomas, this effect can also be observed in other rapidly growing lesions (16,18).

In this study, the majority of osteosarcomas were histologically classified as osteoblastic, two cases presented a chondroblastic matrix, and no fibroblastic matrix was observed. Other studies reported the prevalence of osteoblastic osteosarcomas in the mandible (14,19), albeit the clinical relevance of this histological classification is still controversial. Although chondroblastic osteosarcomas seem to correlate to a better overall survival rate, the prognostic value of this matrix type requires further validation (20).

This retrospective cross-sectional observational study presents inherent method limitations such as the lack of some details not reported by clinicians when requesting the exam as well as the impossibility of clinical follow-up to evaluate factors related to the patient’s prognosis and survival. Therefore, further longitudinal studies are encouraged.

In conclusion, osteosarcomas of the jaws are rare lesions that mainly affect the mandibular body and are characterized by osteoblastic matrix, rapid evolution, and swollen and reddish ulcerated areas. Imaging exams usually reveal a mixed lesion that destructs/invades adjacent structures and causes tooth displacement and sunray periosteal reaction.

## Figures and Tables

**Table 1:** Clinical and histopathological information of the osteosarcomas cases.

Case	Gender	Age	Origin	Evolution (months)	Pain	Clinical appearance	Biopsy	Location	Histology
1	Male	53	Brazil	6	Yes	Swollen and reddish area	Incisional	Maxilla	Osteoblastic
2	Male	18	Brazil	1.5	No	Swollen and reddish area	Incisional	Mandible	Condroblastic
3	Female	81	Brazil	2	No	Ulceration	Incisional	Mandible	Condroblastic
4	Male	49	Brazil	3	Yes	Swollen, reddish, and ulcerated area	Incisional	Mandible	Osteoblastic
5	Male	16	Brazil	2	Yes	Swollen, reddish, and ulcerated area	Incisional	Mandible	Osteoblastic
6	Male	63	N.R.	N.R.	N.R.	Swollen, reddish, and ulcerated area	Not informed	Mandible	Osteoblastic
7	Female	13	Angola	N.R.	N.R.	Swollen, reddish, and ulcerated area	Incisional	Mandible	Osteoblastic
8	Female	33	Brazil	21	N.R.	Swollen, reddish, and ulcerated area	Incisional	Mandible	Osteoblastic
9	Female	30	Brazil	1	Yes	Not informed	Incisional	Maxilla	Osteoblastic
10	Male	22	Brazil	N.R.	N.R.	Not informed	Incisional	Maxilla	Osteoblastic

N.R.: not reported.

**Table 2:** Radiographic appearance of the osteosarcoma lesions.

Case	Imaging exam	Internal aspect	Margins	Teeth	Pericementum	Mandibular canal	Cortical bone	Periosteal reaction
1	Panoramic	Radiopacity	ill-defined	Absent	Not evaluated	Not involved	Erosion	Sunray
2	Panoramic	Radiopacity	ill-defined	Normal	Thickening	Erosion	Normal	Sunray
3	Panoramic	Mixed	ill-defined	Resorption	Normal	Erosion	Normal	Sunray
4	Panoramic and occlusal	Radiopacity	ill-defined	Displaced	Normal	Erosion	Erosion	Sunray
5	Panoramic	Radiolucency	ill-defined	Displaced	Thickening	Normal	Normal	Absent
6	Panoramic and CBCT	Mixed	ill-defined	Resorption	Normal	Displaced	Erosion and expansion	Sunray
7	Panoramic, CT, and teleradiograph	Mixed	ill-defined	Displaced	Thickening	Erosion	Erosion	Sunray
8	Panoramic	Mixed	ill-defined	Resorption	Thickening	Erosion	Erosion	Sunray
9	CBCT	Mixed	ill-defined	Resorption	Thickening	Not involved	Erosion	Sunray
10	Panoramic	Mixed	ill-defined	Normal	Normal	Not involved	Erosion	Absent

CBCT: cone beam computed tomography; TC: multislice computed tomography.

## Data Availability

The datasets used and/or analyzed during the current study are available from the corresponding author.
